# μ_4_-Orthothio­carbonato-tetra­kis­[tri­carbonyl­iron(I)](2 *Fe*—*Fe*)

**DOI:** 10.1107/S1600536811041936

**Published:** 2011-10-12

**Authors:** Yao-Cheng Shi, Huan-Ren Cheng, Li-Min Yuan, Qian-Kun Li

**Affiliations:** a College of Chemistry and Chemical Engineering, Yangzhou University, Yangzhou 225002, People’s Republic of China; b Testing Center, Yangzhou University, Yangzhou 225009, People’s Republic of China; c Hubei Research Institue of Geophysics Survey and Design, Wuhan 430056, People’s Republic of China

## Abstract

The fused bis-butterfly-shaped title compound, [Fe_4_(CS_4_)(CO)_12_], possesses an orthothio­carbonate (CS_4_^4−^) ligand that acts as a bridge between two Fe_2_(CO)_6_ units. A short intra­molecular S⋯S contact [2.6984 (8) and 2.6977 (8) Å] occurs in each S_2_Fe_2_(CO)_6_ fragment.

## Related literature

For general background to related complexes, see: Mathur *et al.* (2009 ▶). For uses of *R*_3_P/CS_2_ in coordination chemistry and organometallic chemistry, see: Galindo *et al.* (1999 ▶). For the synthesis of butterfly S_2_Fe_2_(CO)_6_ complexes, see: Song (2005 ▶). For related structures, see: Shaver *et al.* (1979 ▶); Ortega-Alfaro *et al.* (2004 ▶).
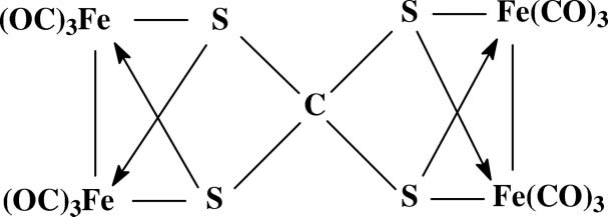


## Experimental

### 

#### Crystal data

[Fe_4_(CS_4_)(CO)_12_]
*M* *_r_* = 699.81
Triclinic, 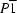

*a* = 9.0875 (9) Å
*b* = 10.9002 (11) Å
*c* = 12.6448 (13) Å
α = 101.8859 (12)°
β = 92.4964 (12)°
γ = 110.0857 (12)°
*V* = 1142.2 (2) Å^3^
*Z* = 2
Mo *K*α radiation
μ = 2.91 mm^−1^
*T* = 296 K
0.15 × 0.12 × 0.11 mm

#### Data collection

Bruker SMART APEX CCD diffractometer
Absorption correction: multi-scan (*SADABS*; Sheldrick, 2004 ▶) *T*_min_ = 0.658, *T*_max_ = 0.721
10006 measured reflections
5128 independent reflections
4237 reflections with *I* > 2σ(*I*)
*R*_int_ = 0.025

#### Refinement

*R*[*F* ^2^ > 2σ(*F* ^2^)] = 0.026
*wR*(*F* ^2^) = 0.070
*S* = 1.04
5128 reflections
298 parameters
6 restraints
Δρ_max_ = 0.35 e Å^−3^
Δρ_min_ = −0.27 e Å^−3^

### 

Data collection: *SMART* (Bruker, 2002 ▶); cell refinement: *SAINT-Plus* (Bruker, 2003 ▶); data reduction: *SAINT-Plus*; program(s) used to solve structure: *SIR2004* (Burla *et al.*, 2005 ▶); program(s) used to refine structure: *SHELXTL* (Sheldrick, 2008 ▶); molecular graphics: *PLATON* (Spek, 2009 ▶) and *WinGX* (Farrugia, 1999 ▶); software used to prepare material for publication: *publCIF* (Westrip, 2010 ▶).

## Supplementary Material

Crystal structure: contains datablock(s) I, global. DOI: 10.1107/S1600536811041936/ng5239sup1.cif

Structure factors: contains datablock(s) I. DOI: 10.1107/S1600536811041936/ng5239Isup2.hkl

Additional supplementary materials:  crystallographic information; 3D view; checkCIF report

## Figures and Tables

**Table:** 

C13—S1	1.827 (2)
C13—S2	1.8300 (19)
C13—S3	1.830 (2)
C13—S4	1.837 (2)
Fe1—S1	2.2730 (6)
Fe1—S2	2.2688 (7)
Fe1—Fe2	2.4949 (5)
Fe2—S1	2.2723 (6)
Fe2—S2	2.2685 (7)
Fe3—S3	2.2676 (6)
Fe3—S4	2.2680 (6)
Fe3—Fe4	2.5007 (5)
Fe4—S3	2.2712 (7)
Fe4—S4	2.2626 (6)

**Table:** 

S1—C13—S2	95.10 (10)
S3—C13—S4	94.73 (9)

**Fig. 1.**
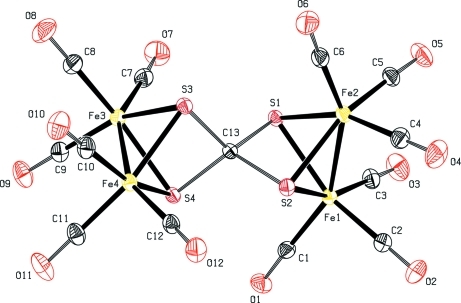
The molecule of the title compound, showing the atom-labelling scheme. Displacement ellipsoids are drawn at the 30% probability level.

## supplementary crystallographic information

### Comment

The activation and cleavage of selected bonds of small molecules by transition metal complexes is one of the challenging subjects of recent researches. CS_2_ has been shown to undergo a variety of reactions with transition metals, including insertion and disproportionation, and there is a growing interest in the activation of CS_2_ from catalytic and biological points of view. The cleavage of the C—S bonds is often observed in various transition metal complexes in which chemistry has been explored for the hydrosulfurization of fossil products. In these complexes, the S^2-^ ion derived from the C—S bond scission functions as a bridging ligand to link metal ions and metal cluster fragments and is generally of use in various cluster growth processes (Mathur *et al.*, 2009).

Interestingly, the reaction of Et_3_P/CS_2_ and Fe_3_(CO)_12_ in THF under inert atmosphere at room temperature leads to the formation of a novel complex (Scheme 1). The molecular structure of the novel complex (Fig. 1) consists of two butterfly Fe_2_(CO)_6_ units connected by a bridging CS_4_ ligand in axial C—S bond fashions similar to the related complex Fe_2_(CO)_6_(µ-S)_2_CH_2_ (Shaver *et al.*, 1979). The Fe—Fe bond lengths are 2.4949 (5) and 2.5007 (5) Å and close to 2.485 (1) Å in Fe_2_(CO)_6_(µ-S)_2_CH_2_, but slightly shorter than 2.511 (1) Å in the complex Fe_2_(CO)_6_(µ-SCH_3_)_2_ (Table 1) (Ortega-Alfaro *et al.*, 2004), the corresponding C—S bond lengths are 1.827 (2), 1.830 (2) and 1.830 (2), 1.837 (2)°, respectively, which are longer than those in the complex Fe_2_(CO)_6_(µ-SCH_3_)_2_. For each S_2_Fe_2_(CO)_6_ butterfly core, the S—C—S bond angle is 95.10 (10) and 94.73 (9)° and close to 94.55 (3)° in Fe_2_(CO)_6_(µ-S)_2_CH_2_ (Table 1). As compared with 2.744 (1)–2.773 (1) Å in Fe_2_(CO)_6_(µ-SCH_3_)_2_, the S···S distance (2.6984 (8) and 2.6977 (8) Å) indicates an intramolecular short contact in each S_2_Fe_2_(CO)_6_ butterfly core.

### Experimental

A THF solution of Et_3_P/CS_2_ (1 mmol) and Fe_3_(CO)_12_ (1 mmol) under inert atmosphere is stirred for 24 h at room temperature. After removal of the solvent, the mixture was purified by chromatography on silica gel with dichloromethane-petroleum ether (*v*/*v*, 1:3) as eluant to give the red-orange solid. Single crystals were grown from ether solution of the title compound.

### Crystal data

**Table:**

[Fe_4_(CS_4_)(CO)_12_]	*Z* = 2
*M**_r_* = 699.81	*F*(000) = 684
Triclinic, *P*1	*D*_x_ = 2.035 Mg m^−^^3^
*a* = 9.0875 (9) Å	Mo *K*α radiation, λ = 0.71073 Å
*b* = 10.9002 (11) Å	Cell parameters from 4237 reflections
*c* = 12.6448 (13) Å	θ = 1.7–27.5°
α = 101.8859 (12)°	µ = 2.91 mm^−^^1^
β = 92.4964 (12)°	*T* = 296 K
γ = 110.0857 (12)°	Prism, red
*V* = 1142.2 (2) Å^3^	0.15 × 0.12 × 0.11 mm

### Data collection

**Table:**

Bruker SMART APEX CCD diffractometer	5128 independent reflections
Radiation source: fine-focus sealed tube	4237 reflections with *I* > 2σ(*I*)
graphite	*R*_int_ = 0.025
ω and φ scans	θ_max_ = 27.5°, θ_min_ = 1.7°
Absorption correction: multi-scan (*SADABS*; Sheldrick, 2004)	*h* = −11→11
*T*_min_ = 0.658, *T*_max_ = 0.721	*k* = −14→14
10006 measured reflections	*l* = −16→16

### Refinement

**Table:**

Refinement on *F*^2^	6 restraints
Least-squares matrix: full	Primary atom site location: structure-invariant direct methods
*R*[*F*^2^ > 2σ(*F*^2^)] = 0.026	Secondary atom site location: difference Fourier map
*wR*(*F*^2^) = 0.070	*w* = 1/[σ^2^(*F*_o_^2^) + (0.0332*P*)^2^] where *P* = (*F*_o_^2^ + 2*F*_c_^2^)/3
*S* = 1.04	(Δ/σ)_max_ < 0.001
5128 reflections	Δρ_max_ = 0.35 e Å^−^^3^
298 parameters	Δρ_min_ = −0.27 e Å^−^^3^

### Special details

**Table:**

Geometry. All e.s.d.'s (except the e.s.d. in the dihedral angle between two l.s. planes) are estimated using the full covariance matrix. The cell e.s.d.'s are taken into account individually in the estimation of e.s.d.'s in distances, angles and torsion angles; correlations between e.s.d.'s in cell parameters are only used when they are defined by crystal symmetry. An approximate (isotropic) treatment of cell e.s.d.'s is used for estimating e.s.d.'s involving l.s. planes.
Refinement. Refinement of *F*^2^ against ALL reflections. The weighted *R*-factor *wR* and goodness of fit *S* are based on *F*^2^, conventional *R*-factors *R* are based on *F*, with *F* set to zero for negative *F*^2^. The threshold expression of *F*^2^ > σ(*F*^2^) is used only for calculating *R*-factors(gt) *etc*. and is not relevant to the choice of reflections for refinement. *R*-factors based on *F*^2^ are statistically about twice as large as those based on *F*, and *R*- factors based on ALL data will be even larger.

### Fractional atomic coordinates and isotropic or equivalent isotropic displacement parameters (Å^2^)

**Table:**

	*x*	*y*	*z*	*U*_iso_*/*U*_eq_	
C1	0.6561 (3)	0.7935 (2)	0.77984 (18)	0.0440 (5)	
C2	0.8526 (3)	0.8863 (3)	0.6347 (2)	0.0506 (6)	
C3	0.9578 (3)	0.9654 (3)	0.8400 (2)	0.0495 (6)	
C4	1.1219 (3)	0.7705 (3)	0.5832 (2)	0.0576 (7)	
C5	1.2260 (3)	0.8567 (3)	0.7869 (2)	0.0499 (6)	
C6	1.1533 (3)	0.5863 (3)	0.6883 (2)	0.0520 (6)	
C7	0.7715 (3)	0.4656 (3)	1.0044 (2)	0.0518 (6)	
C8	0.6869 (3)	0.2015 (3)	0.9062 (2)	0.0571 (7)	
C9	0.4644 (3)	0.2955 (2)	0.94124 (17)	0.0461 (6)	
C10	0.5449 (3)	0.1072 (3)	0.6532 (2)	0.0533 (6)	
C11	0.3306 (3)	0.2117 (3)	0.69401 (19)	0.0538 (6)	
C12	0.5320 (3)	0.3000 (2)	0.54991 (19)	0.0461 (5)	
C13	0.7801 (2)	0.5349 (2)	0.75626 (15)	0.0326 (4)	
Fe1	0.85275 (4)	0.80727 (3)	0.74597 (2)	0.03532 (9)	
Fe2	1.06610 (4)	0.71578 (3)	0.70503 (2)	0.03858 (9)	
Fe3	0.64765 (4)	0.34558 (3)	0.88425 (2)	0.03604 (9)	
Fe4	0.54035 (4)	0.27424 (3)	0.68685 (2)	0.03649 (9)	
O1	0.5347 (2)	0.7883 (2)	0.80095 (16)	0.0652 (5)	
O2	0.8523 (3)	0.9343 (2)	0.56303 (17)	0.0808 (7)	
O3	1.0252 (3)	1.0659 (2)	0.89976 (17)	0.0801 (6)	
O4	1.1562 (3)	0.8060 (3)	0.50561 (17)	0.0906 (8)	
O5	1.3234 (2)	0.9495 (2)	0.83931 (18)	0.0764 (6)	
O6	1.2101 (3)	0.5078 (2)	0.67522 (19)	0.0802 (6)	
O7	0.8437 (3)	0.5364 (2)	1.08183 (16)	0.0849 (7)	
O8	0.7108 (3)	0.1096 (2)	0.9194 (2)	0.0923 (7)	
O9	0.3472 (2)	0.2622 (2)	0.97500 (15)	0.0716 (6)	
O10	0.5481 (3)	0.0019 (2)	0.63331 (18)	0.0840 (7)	
O11	0.1982 (3)	0.1726 (3)	0.69931 (18)	0.0901 (7)	
O12	0.5245 (2)	0.3122 (2)	0.46352 (14)	0.0717 (6)	
S1	0.92568 (6)	0.68363 (5)	0.84778 (4)	0.03454 (12)	
S2	0.80928 (7)	0.60300 (6)	0.63482 (4)	0.03789 (13)	
S3	0.80213 (6)	0.37272 (5)	0.74843 (4)	0.03674 (12)	
S4	0.57807 (6)	0.47963 (5)	0.79267 (4)	0.03420 (12)	

### Atomic displacement parameters (Å^2^)

**Table:**

	*U*^11^	*U*^22^	*U*^33^	*U*^12^	*U*^13^	*U*^23^
C1	0.0464 (14)	0.0398 (13)	0.0478 (12)	0.0174 (11)	0.0032 (10)	0.0116 (10)
C2	0.0482 (15)	0.0460 (14)	0.0586 (14)	0.0159 (12)	0.0062 (12)	0.0168 (12)
C3	0.0446 (14)	0.0430 (14)	0.0568 (14)	0.0113 (12)	0.0098 (11)	0.0103 (12)
C4	0.0577 (17)	0.0620 (17)	0.0653 (17)	0.0292 (14)	0.0259 (14)	0.0241 (14)
C5	0.0368 (14)	0.0484 (15)	0.0656 (16)	0.0140 (12)	0.0142 (12)	0.0163 (13)
C6	0.0452 (15)	0.0523 (15)	0.0623 (15)	0.0179 (12)	0.0168 (12)	0.0189 (13)
C7	0.0536 (16)	0.0528 (15)	0.0469 (13)	0.0150 (13)	0.0004 (11)	0.0155 (12)
C8	0.0601 (17)	0.0545 (16)	0.0633 (16)	0.0241 (14)	0.0094 (13)	0.0218 (13)
C9	0.0510 (15)	0.0453 (14)	0.0360 (11)	0.0114 (12)	0.0063 (10)	0.0065 (10)
C10	0.0601 (17)	0.0402 (14)	0.0502 (14)	0.0095 (12)	0.0063 (12)	0.0057 (11)
C11	0.0481 (16)	0.0526 (16)	0.0440 (13)	0.0020 (13)	0.0040 (11)	0.0040 (11)
C12	0.0436 (14)	0.0401 (13)	0.0434 (13)	0.0046 (11)	0.0000 (10)	0.0049 (10)
C13	0.0291 (11)	0.0319 (10)	0.0341 (10)	0.0089 (9)	0.0031 (8)	0.0057 (8)
Fe1	0.03283 (18)	0.03237 (17)	0.03938 (17)	0.00973 (13)	0.00397 (13)	0.00912 (13)
Fe2	0.03253 (18)	0.03943 (18)	0.04416 (18)	0.01127 (14)	0.01067 (13)	0.01241 (14)
Fe3	0.03703 (18)	0.03452 (17)	0.03534 (16)	0.01093 (14)	0.00442 (13)	0.00903 (13)
Fe4	0.03603 (18)	0.03052 (17)	0.03559 (16)	0.00580 (13)	0.00263 (13)	0.00313 (13)
O1	0.0438 (11)	0.0676 (13)	0.0955 (14)	0.0303 (10)	0.0199 (10)	0.0237 (11)
O2	0.0919 (17)	0.0883 (16)	0.0771 (13)	0.0311 (14)	0.0146 (12)	0.0530 (13)
O3	0.0750 (15)	0.0484 (12)	0.0894 (15)	0.0052 (11)	0.0037 (12)	−0.0119 (11)
O4	0.112 (2)	0.112 (2)	0.0796 (14)	0.0537 (16)	0.0579 (14)	0.0566 (15)
O5	0.0456 (12)	0.0589 (13)	0.1025 (16)	0.0024 (10)	0.0007 (11)	0.0014 (12)
O6	0.0805 (16)	0.0736 (15)	0.1125 (17)	0.0495 (13)	0.0371 (13)	0.0347 (13)
O7	0.0857 (16)	0.0882 (16)	0.0530 (11)	0.0075 (13)	−0.0226 (11)	0.0045 (11)
O8	0.1114 (19)	0.0722 (15)	0.1227 (19)	0.0545 (15)	0.0225 (15)	0.0475 (15)
O9	0.0573 (13)	0.0795 (15)	0.0636 (12)	0.0077 (11)	0.0265 (10)	0.0115 (11)
O10	0.1131 (19)	0.0391 (11)	0.0960 (16)	0.0283 (12)	0.0166 (14)	0.0056 (11)
O11	0.0473 (13)	0.1058 (19)	0.0861 (15)	−0.0053 (12)	0.0126 (11)	0.0119 (14)
O12	0.0788 (14)	0.0732 (14)	0.0428 (10)	0.0033 (11)	−0.0061 (9)	0.0147 (9)
S1	0.0314 (3)	0.0334 (3)	0.0332 (2)	0.0058 (2)	0.0010 (2)	0.0063 (2)
S2	0.0398 (3)	0.0375 (3)	0.0317 (3)	0.0086 (2)	0.0031 (2)	0.0077 (2)
S3	0.0338 (3)	0.0338 (3)	0.0430 (3)	0.0135 (2)	0.0070 (2)	0.0071 (2)
S4	0.0298 (3)	0.0309 (3)	0.0400 (3)	0.0097 (2)	0.0045 (2)	0.0062 (2)

### Geometric parameters (Å, °)

**Table:**

C1—O1	1.131 (3)	C10—Fe4	1.798 (3)
C1—Fe1	1.819 (3)	C11—O11	1.140 (3)
C2—O2	1.136 (3)	C11—Fe4	1.804 (3)
C2—Fe1	1.795 (2)	C12—O12	1.129 (3)
C3—O3	1.142 (3)	C12—Fe4	1.813 (2)
C3—Fe1	1.796 (3)	C13—S1	1.827 (2)
C4—O4	1.145 (3)	C13—S2	1.8300 (19)
C4—Fe2	1.795 (3)	C13—S3	1.830 (2)
C5—O5	1.142 (3)	C13—S4	1.837 (2)
C5—Fe2	1.795 (3)	Fe1—S1	2.2730 (6)
C6—O6	1.130 (3)	Fe1—S2	2.2688 (7)
C6—Fe2	1.824 (3)	Fe1—Fe2	2.4949 (5)
C7—O7	1.127 (3)	Fe2—S1	2.2723 (6)
C7—Fe3	1.818 (3)	Fe2—S2	2.2685 (7)
C8—O8	1.138 (3)	Fe3—S3	2.2676 (6)
C8—Fe3	1.796 (3)	Fe3—S4	2.2680 (6)
C9—O9	1.134 (3)	Fe3—Fe4	2.5007 (5)
C9—Fe3	1.798 (3)	Fe4—S3	2.2712 (7)
C10—O10	1.135 (3)	Fe4—S4	2.2626 (6)
			
S1···S2	2.6984 (8)	S3···S4	2.6977 (8)
			
O1—C1—Fe1	178.3 (2)	C6—Fe2—Fe1	154.51 (8)
O2—C2—Fe1	178.8 (2)	S2—Fe2—Fe1	56.649 (18)
O3—C3—Fe1	179.6 (3)	S1—Fe2—Fe1	56.723 (17)
O4—C4—Fe2	179.2 (3)	C8—Fe3—C9	91.66 (12)
O5—C5—Fe2	177.0 (2)	C8—Fe3—C7	97.13 (12)
O6—C6—Fe2	177.6 (2)	C9—Fe3—C7	98.48 (11)
O7—C7—Fe3	176.7 (2)	C8—Fe3—S3	93.97 (9)
O8—C8—Fe3	179.5 (3)	C9—Fe3—S3	155.51 (7)
O9—C9—Fe3	178.5 (2)	C7—Fe3—S3	104.42 (8)
O10—C10—Fe4	179.2 (3)	C8—Fe3—S4	158.63 (9)
O11—C11—Fe4	179.5 (3)	C9—Fe3—S4	93.86 (8)
O12—C12—Fe4	178.0 (2)	C7—Fe3—S4	102.43 (8)
S1—C13—S3	118.10 (10)	S3—Fe3—S4	72.99 (2)
S1—C13—S2	95.10 (10)	C8—Fe3—Fe4	102.35 (9)
S3—C13—S2	117.13 (11)	C9—Fe3—Fe4	98.88 (7)
S1—C13—S4	116.95 (11)	C7—Fe3—Fe4	153.39 (8)
S3—C13—S4	94.73 (9)	S3—Fe3—Fe4	56.635 (18)
S2—C13—S4	116.62 (10)	S4—Fe3—Fe4	56.394 (17)
C2—Fe1—C3	92.20 (12)	C10—Fe4—C11	92.11 (13)
C2—Fe1—C1	97.54 (11)	C10—Fe4—C12	97.98 (11)
C3—Fe1—C1	96.81 (11)	C11—Fe4—C12	97.54 (11)
C2—Fe1—S2	93.44 (9)	C10—Fe4—S4	157.10 (8)
C3—Fe1—S2	158.89 (8)	C11—Fe4—S4	93.60 (9)
C1—Fe1—S2	102.58 (8)	C12—Fe4—S4	103.21 (8)
C2—Fe1—S1	156.74 (8)	C10—Fe4—S3	94.03 (9)
C3—Fe1—S1	94.58 (8)	C11—Fe4—S3	157.73 (8)
C1—Fe1—S1	103.69 (7)	C12—Fe4—S3	102.76 (8)
S2—Fe1—S1	72.90 (2)	S4—Fe4—S3	73.03 (2)
C2—Fe1—Fe2	100.11 (8)	C10—Fe4—Fe3	100.52 (8)
C3—Fe1—Fe2	102.33 (8)	C11—Fe4—Fe3	101.33 (8)
C1—Fe1—Fe2	153.30 (7)	C12—Fe4—Fe3	152.93 (8)
S2—Fe1—Fe2	56.636 (18)	S4—Fe4—Fe3	56.600 (16)
S1—Fe1—Fe2	56.695 (17)	S3—Fe4—Fe3	56.499 (16)
C5—Fe2—C4	91.26 (13)	C13—S1—Fe2	88.02 (6)
C5—Fe2—C6	100.64 (12)	C13—S1—Fe1	87.12 (6)
C4—Fe2—C6	96.91 (11)	Fe2—S1—Fe1	66.582 (18)
C5—Fe2—S2	153.65 (8)	C13—S2—Fe2	88.06 (7)
C4—Fe2—S2	93.88 (9)	C13—S2—Fe1	87.17 (7)
C6—Fe2—S2	104.35 (9)	Fe2—S2—Fe1	66.715 (19)
C5—Fe2—S1	93.23 (8)	C13—S3—Fe3	87.77 (7)
C4—Fe2—S1	157.61 (9)	C13—S3—Fe4	87.52 (7)
C6—Fe2—S1	103.77 (8)	Fe3—S3—Fe4	66.867 (19)
S2—Fe2—S1	72.92 (2)	C13—S4—Fe4	87.60 (7)
C5—Fe2—Fe1	97.00 (8)	C13—S4—Fe3	87.58 (6)
C4—Fe2—Fe1	100.96 (8)	Fe4—S4—Fe3	67.006 (19)
